# Takotsubo cardiomyopathy following ultrasound-guided renal cyst aspiration

**DOI:** 10.1259/bjrcr.20180101

**Published:** 2019-01-03

**Authors:** Dorissa Lahner Gursahaney, Dominik M Wiktor, Jonathan Lindquist

**Affiliations:** 1 Department of Radiology, School of Medicine, University of Colorado, Aurora, CO, USA; 2 Department of Cardiovascular Medicine, University of Colorado School of Medicine, Aurora, CO, USA

## Abstract

Interventional radiology plays a critical role in offering minimally invasive procedures, resulting in increased patient comfort. However, of the millions of patients undergoing interventional procedures each year, many suffer from pre-procedural psychological stressors related to fear of discomfort and diagnostic uncertainty. We describe a case of Takotsubo cardiomyopathy, also called broken heart syndrome or stress cardiomyopathy, following ultrasound-guided renal cyst aspiration in a patient with severe anxiety in anticipation of the interventional radiology procedure.

## Case presentation

**Figure 1.**
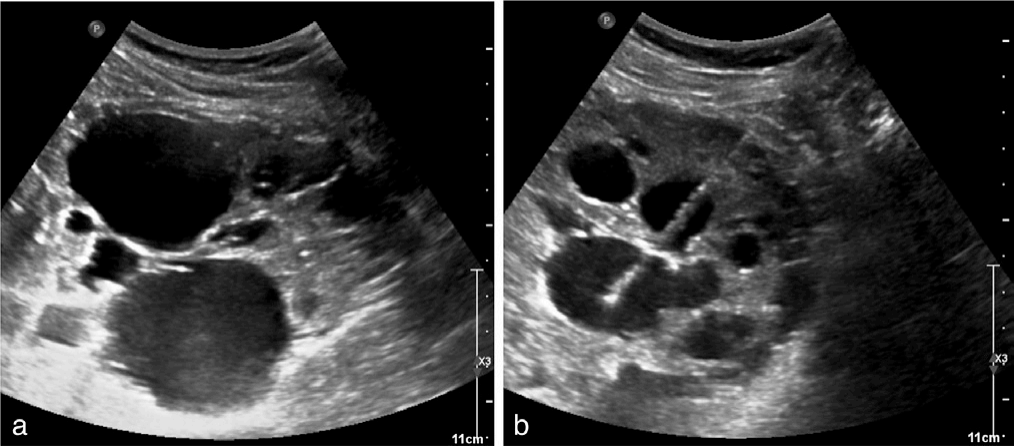
(a–b) A 48-year-old healthy female with history of ADPKD presents for routine outpatient renal cyst aspiration. Ultrasound of the right kidney (a) demonstrates multiple renal cysts. Renal cysts were aspirated under ultrasound guidance (b) using an 18-gauge Chiba needle. ADPKD, autosomal dominant polycystic kidney disease.

A 48-year-old healthy female with a history of autosomal dominant polycystic kidney disease (ADPKD), premature ventricular contractions, hypertension, hyperlipidaemia, and anxiety presented for elective outpatient right renal cyst aspiration for right upper quadrant abdominal pain secondary to renal capsular stretch. Her family history is pertinent for a father with ADPKD and several myocardial infarctions beginning in his forties. The patient was seen in interventional radiology clinic. The procedure was explained to the patient in clinic and written informed consent was obtained. The patient presented for her procedure 2 weeks after her clinic visit. After administration of 1% lidocaine local anaesthetic and intravenous moderate sedation, an 18-gauge Chiba needle was advanced into multiple right renal cysts under ultrasound guidance (Figure 1). A total of 170 ml of clear yellow fluid was aspirated. The patient reported symptomatic improvement and remained in stable condition in the interventional radiology suite. There was no procedural complication, uncontrolled pain, or immediate complication encountered. One hour post-procedure, she complained of severe abdominal pain. Physical examination revealed a soft abdomen and cool dry skin. Vital signs were notable for bradycardia and hypotension. An intravenous fluid bolus and Trendelenberg positioning was initiated immediately, however, the patient rapidly declined and became unresponsive with undetectable pulse.

## Investigations

**Figure 2.**
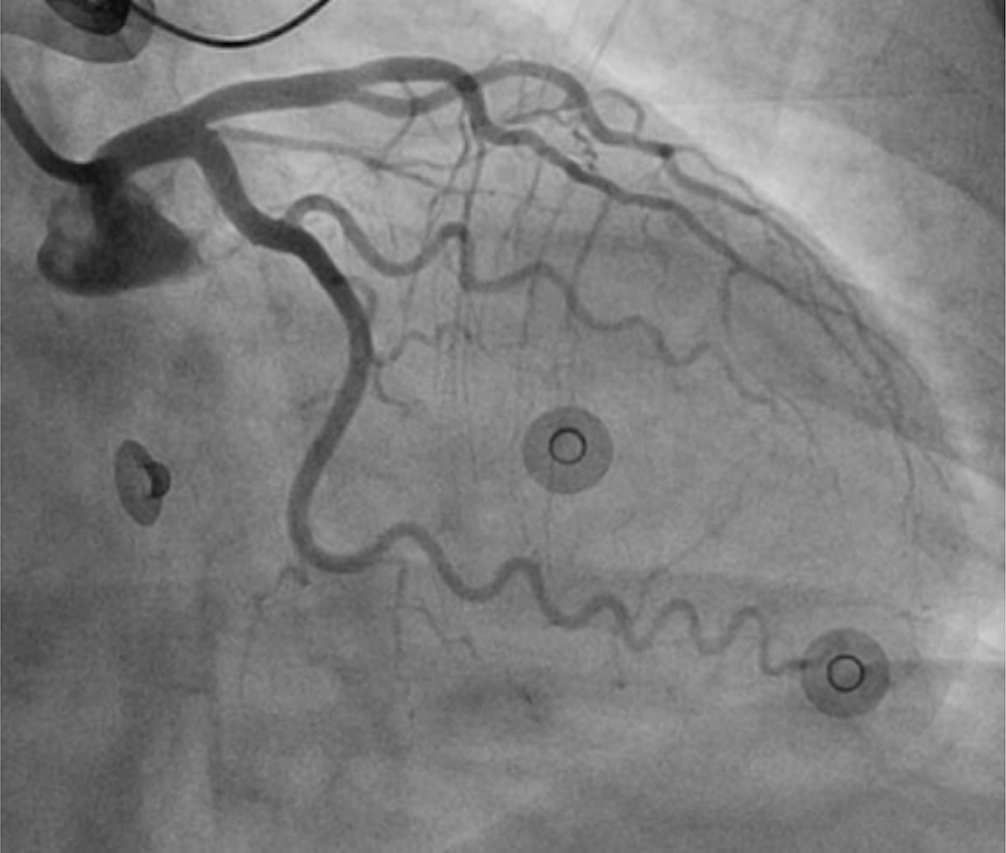
Left coronary angiography. The left anterior descending and circumflex coronary arteries have no angiographically apparent coronary atherosclerosis, spasm or embolic occlusion.

**Figure 3.**
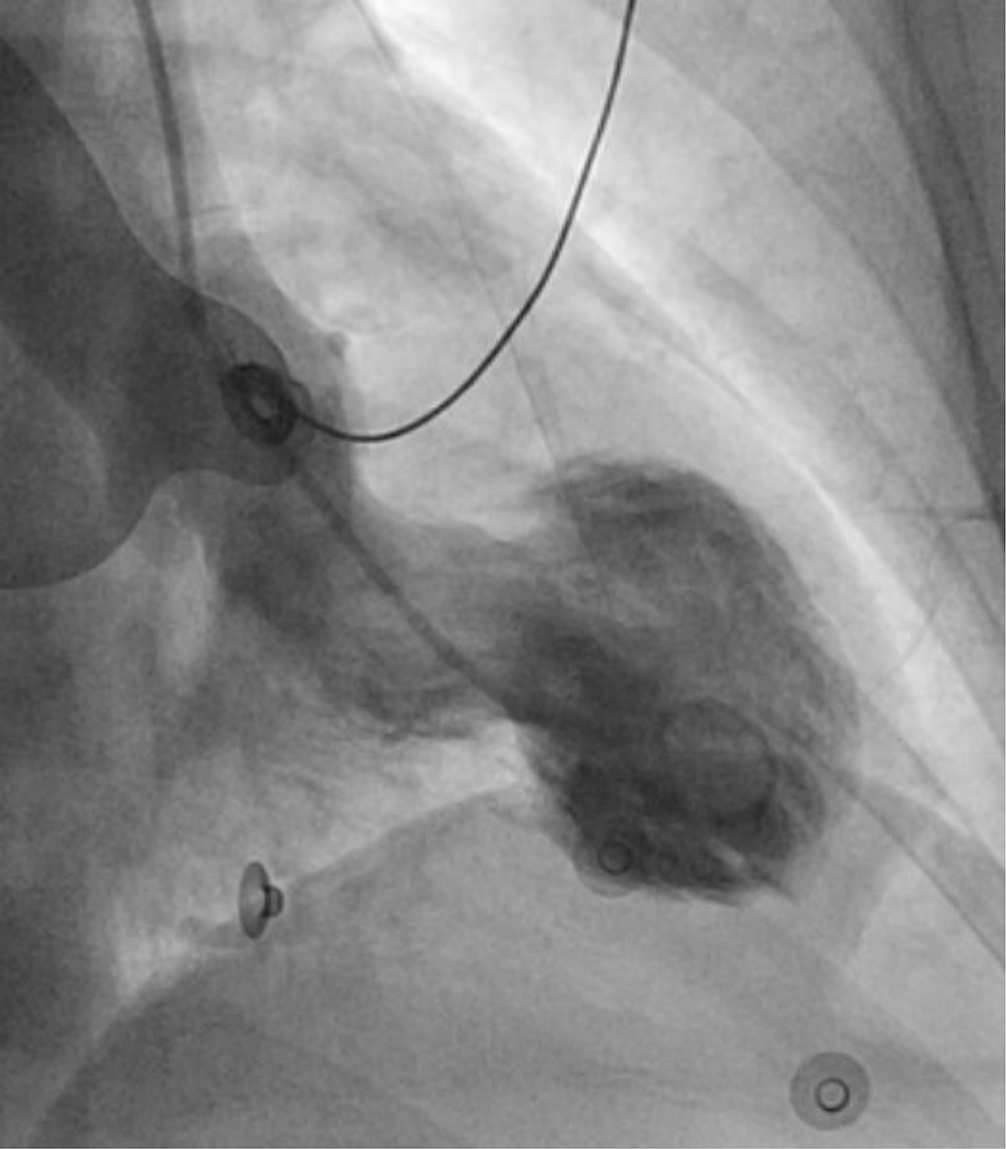
Left ventriculogram in systole reveals apical ballooning due to severe hypokinesis in the apical segments and hyperdynamic basal segments, consistent with Takotsubo cardiomyopathy.

Radiologists at bedside initiated a medical emergency code for cardiac arrest, began cardiopulmonary resuscitation, and administered intravenous epinephrine. Upon return of spontaneous circulation and improved responsiveness, the patient endorsed severe chest pain. Bedside ultrasound showed no right flank haematoma or pericardial effusion. EKG revealed ST elevations in anterolateral leads and depressions in inferior leads suspicious for ST-elevation myocardial infarction. A chest radiograph was normal. Labs revealed normal initial troponin, serum glucose, and serum potassium levels. The patient was taken emergently to the cardiac catheterization lab where coronary angiography demonstrated angiographically normal coronary arteries (Figure 2). Left ventriculography revealed apical segment akinesis with hyperdynamic basal segments, and left ventricular systolic function of 45% consistent with Takotsubo cardiomyopathy (TTC) (Figure 3). Discussions with the patient and her family at bedside revealed that she suffers from anxiety at baseline and experienced severe anxiety regarding the renal cyst aspiration for several weeks prior to the procedure. There were no additional indicators that the patient would react to the procedure with severe anxiety, as she appeared comfortable during the periprocedural period. She reported no prior procedures.

## Differential diagnosis

Abdominal pain and haemodynamic instability following renal intervention is most suspicious for post-procedural haemorrhage, particularly in a patient with a history of hypertension. However, compensatory tachycardia would be expected rather than the patient’s bradycardia, which was the earliest indication of a different underlying aetiology. The development of cardiovascular collapse and severe chest pain, even though it followed cardiopulmonary resuscitation, necessitated prompt work-up of a broad differential. Differential diagnosis included acute coronary syndrome, cardiac tamponade, pneumothorax, pulmonary embolus, pulmonary hypertension, and metabolic abnormalities such as hypoglycaemia or potassium derangements among others. The patient’s electrocardiogram findings, cardiac risk factors, and family history of MI narrowed the differential to a cardiogenic aetiology with ST-elevation myocardial infarction considered most likely. TTC was not specifically suspected prior to definitive diagnosis with left ventriculography.

## Treatment

The patient was admitted to the intensive care unit for supportive care including close monitoring of volume status and blood pressure, trending troponin levels, repeat electrocardiogram, and telemetry. A transthoracic echocardiogram revealed no left ventricular thrombus, normalization of wall motion, and improved left ventricular function with ejection fraction estimated at 55–60%. Troponin levels peaked at 4.92 ng ml^−1^. Prior to discharge the patient was restarted on home medications including metoprolol and atorvastatin. She was discharged home on hospital Day 6 with outpatient cardiology follow-up.

## Outcome and follow-up

Upon outpatient follow-up in cardiology clinic 8 weeks after hospital discharge, the patient remained asymptomatic from a cardiac standpoint. A repeat electrocardiogram at this time was normal and a stress echocardiogram demonstrated normal left ventricular size and systolic function. The patient endorsed ongoing treatment of anxiety.

## Discussion

TTC is a transient, acute cardiomyopathy characterized by left ventricular wall motion abnormalities in the absence of coronary artery occlusion.^1,2^ It was first described in Japan in 1990 and named after the “Takotsubo”, a ceramic octopus trap that bears a striking resemblance to the abnormal contours of the left ventricle in systole.^3^ Clinical presentation often mimics acute coronary syndrome and is typically preceded by a profoundly stressful event, such as the loss of a loved one, though other triggers may be more subtle. The pathophysiology underlying TTC is not well established, however stress-induced release of catecholamines, has been proposed.^1,4^ TTC accounts for 1–2% of patients presenting with ACS and is associated with a generally favourable prognosis with hospital mortality of 0–10%.^1,5^ Other stressful precursors, including exercise and intense anger, have been reported as triggering TTC.^4,6^ Since TTC mimics acute coronary syndrome, initial treatment includes supplemental oxygen, aspirin, β blockers, and intravenous heparin with consideration for early coronary angiography, especially with ST-segment elevations on ECG or with ongoing chest discomfort.^1^ Once diagnosed, the condition is generally managed medically with angiotensin-converting-enzyme inhibitors and β blockers.^4^ In severe cases, inotropic support and mechanical circulatory support such as intra-aortic balloon pumping are beneficial.^7^ Apical akinesis resolves in the majority of patients within a month.^1^

There are millions of interventional radiology procedures performed annually and it is well established that many patients experience heightened anxiety, attributed both to anticipated discomfort and possible results.^8–12^ Interventional radiologists find fulfilment, in part, by offering greater patient comfort and minimizing procedural invasiveness. It is therefore of great value for us to recognize patients who may be suffering psychological discomfort related to interventional procedures. While pre-procedure anxiety is very common in patients, female gender, uncertain diagnosis, and undergoing a procedure for the first time have been associated with higher levels of anxiety.^8^ Patients have been shown to benefit from increased psychological preparation to reduce pre-procedure anxiety. Easily adoptable strategies include describing the procedural steps and equipment, outlining the patient’s role in facilitating the intervention and their recovery, discussion of emotions, and relaxation training such as deep breathing.^8^ Furthermore, attention to reducing pre-procedure anxiety may have a positive impact on department resources by decreasing cancellations and missed appointments.^11^

An additional point of relevance at the intersection of radiology and TTC is that of epinephrine. Epinephrine plays an essential role in the treatment of contrast media reactions and is used by radiologists to respond to anaphylaxis, severe bronchospasm or laryngeal oedema, and severe hypotension related to the administration of contrast media. However, epinephrine has been implicated in precipitating TTC in clinical practice.^13^ A systematic review supports the hypothesis that catecholamines, whether exogenous or endogenous, are associated with TTC pathophysiology. Radiologists using epinephrine should be aware of this association and recognize that continued vasopressor administration may result in outflow tract obstruction.^13^

Lastly, this case underscores the importance of rapidly building and investigating a broad differential diagnosis in the setting of post-procedural haemodynamic instability and cardiovascular collapse. Common things being common, the aetiology of hypotension following renal cyst aspiration is renal haemorrhage. However, quickly excluding this with bedside ultrasound while simultaneously evaluating for other aetiologies allowed us to quickly arrive at the correct diagnosis. Had this been an ST-segment elevation myocardial infarction as initially suspected, prompt intervention and reperfusion would have made a tremendous impact on preventing possible morbidity and mortality. Neglecting a broad differential when responding to a post-procedural complication may lead the interventional radiologist to a perilous dead-end, much like the unfortunate octopus into a takotsubo.

## Learning points

1. TTC is a transient, acute cardiomyopathy characterized by left ventricular wall motion abnormalities that can mimic proximal LAD ischaemia/infarction on echocardiography in the absence of coronary artery occlusion. Clinical presentation is typically preceded by a profoundly stressful event; in this case, an interventional radiology procedure in a patient with severe pre-procedural anxiety.
2. Many patients experience heightened anxiety prior to radiology procedures attributed both to anticipated discomfort and possible results. Providing these patients with increased psychological preparation has been shown to reduce pre-procedural anxiety.
3. Epinephrine, a critical drug in the treatment of acute cardiovascular collapse and severe contrast media reactions, has been implicated in precipitating TTC in clinical practice. Radiologists using epinephrine should be aware of this association and know that continued vasopressor administration may result in outflow tract obstruction.
4. Maintaining a broad differential when responding to post-procedural complications in critical to quickly arriving at the correct diagnosis.
